# Multiscale Characterization of Mesh–Tissue Interface Disorganization in Chronic Mesh-Associated Inflammatory Remodeling Using Histology and Atomic Force Microscopy: An Exploratory Study

**DOI:** 10.3390/life16101679

**Published:** 2026-10-07

**Authors:** Adelina Tanevski, Delia Gabriela Ciobanu Apostol, Andreea Ludușanu, Bogdan Mihnea Ciuntu, Ștefan Lucian Toma, Gheorghe Balan, Raoul-Vasile Lupușoru, Ciprian Gavrilă Ilea, Mihaela Tîrnovanu, Bogdan Florin Toma

**Affiliations:** 1 Faculty of Medicine, Grigore T. Popa University of Medicine and Pharmacy, 700115 Iasi, Romania; delia.ciobanu@umfiasi.ro (D.G.C.A.);; 2 Faculty of Materials Science and Engineering, Gheorghe Asachi Technical University of Iași, 700050 Iasi, Romania

**Keywords:** surgical mesh, mesh–tissue interface, abdominal wall repair, chronic mesh-associated inflammation, foreign body response, extracellular matrix remodeling, collagen organization, atomic force microscopy, histopathology, surface roughness

## Abstract

Background/Objectives: Chronic mesh-associated inflammatory remodeling may complicate abdominal wall repair through persistent foreign body response, fibrosis, and extracellular matrix disorganization. This exploratory study compared pathological and well-integrated mesh–tissue interfaces using histology and atomic force microscopy (AFM). Methods: Fourteen mesh–tissue complexes were analyzed: seven pathological explants and seven well-integrated reference specimens. Hematoxylin–eosin and Van Gieson staining assessed inflammation, foreign body reaction, fibrosis, and collagen organization. Contact-mode AFM evaluated surface topography, fibrillar-like organization, directionality, and ISO 25178-2 areal surface parameters. Values from 64 scans per specimen were averaged to obtain one specimen-level value, and groups were compared using the two-sided Mann–Whitney U test. Results: Pathological explants showed greater fibro-inflammatory heterogeneity, reduced collagen continuity, increased surface heterogeneity, discontinuous fibrillar-like features, and reduced structural organization. Sa, Sq, Sku, Sp, Sv, and Sz were higher, whereas Ssk was lower, in the pathological group (U = 0; exact two-sided *p* = 0.000583 for all parameters). Representative directional analysis suggested broader orientation distribution and reduced preferential alignment. Conclusions: Histology and AFM provided complementary morphological information on mesh–tissue remodeling. Larger standardized studies are required before AFM-derived parameters can be assigned diagnostic or prognostic significance.

## 1. Introduction

Synthetic meshes are widely used in abdominal wall reconstruction because they reduce hernia recurrence and provide mechanical reinforcement of weakened or deficient tissues [1,2]. Despite their clinical benefits, mesh implantation may be followed by chronic complications, including persistent pain, infection, fibrosis, mesh contraction, foreign body sensation, and impaired abdominal wall function. In selected patients, these complications may become sufficiently severe to require partial or complete removal of the prosthetic material [1,2].

Mesh-based repair is recommended by current European Hernia Society guidelines for midline incisional hernia and remains a central strategy for abdominal wall reinforcement [3]. Nevertheless, mesh explantation may become necessary in a subset of patients because of infection, chronic pain, fistula or erosion, recurrence, mesh reaction, migration, or other mesh-related complications. The reported frequency and distribution of these indications vary according to hernia type, mesh location, and referral population. In a large cohort of 59,453 patients undergoing elective inpatient open ventral hernia repair with mesh, 2.2% subsequently underwent mesh removal, which was used as a surrogate for clinically significant mesh infection [4]. In a dedicated mesh-removal series, infection was the most frequent indication for abdominal mesh removal, accounting for 43% of cases, whereas pain predominated among pelvic mesh removals [5]. An earlier series of 347 explanted meshes reported recurrence, chronic pain, infection, and fistula formation in approximately 63%, 30%, 21%, and 7% of specimens, respectively [6].

Mesh implantation initiates a foreign body response characterized by macrophage recruitment, multinucleated giant-cell formation, fibroblast activation, and fibrous tissue deposition [7]. The intensity and organization of this response may be influenced by the composition, architecture, porosity, mechanical properties, and degradation characteristics of the implanted material [8,9]. Under favorable conditions, the resulting tissue response contributes to stable incorporation of the prosthetic material into the surrounding tissues. Conversely, persistent inflammatory stimulation, together with unfavorable material and structural characteristics, may contribute to excessive or disorganized fibrosis, irregular tissue deposition, and loss of organized tissue architecture, resulting in an unfavorable mesh–tissue interface [7,8,9].

The extracellular matrix plays a central role in tissue structure, cellular behavior, and the transmission of mechanical forces [10]. Chronic inflammation may modify extracellular matrix turnover, collagen deposition, and tissue organization, producing spatially heterogeneous structural changes [11]. In addition, the composition and architecture of extracellular matrix-based biomaterials can influence cellular adhesion, migration, differentiation, and remodeling responses [12]. Tissue-specific extracellular matrix characteristics are therefore increasingly recognized as relevant parameters in the design and evaluation of biomaterials intended to interact with native tissues [13].

AFM can be combined with optical microscopy to provide complementary information on biological surface structure across different spatial scales [14]. Its high spatial resolution allows the characterization of surface features that cannot be adequately resolved using conventional light microscopy alone.

From a clinical perspective, chronic pain and foreign body sensation remain important long-term concerns following mesh repair of primary ventral hernias [15]. Histopathological studies of chronically infected abdominal wall meshes have also demonstrated persistent inflammation, foreign body giant-cell reaction, fibrosis, and extracellular matrix remodeling at the mesh–tissue interface [16]. Hematoxylin–eosin staining enables the morphological assessment of inflammatory infiltrates, macrophages, foreign body giant cells, vascular structures, and fibro-inflammatory tissue. Collagen-oriented histochemical staining can provide additional information on fibrosis, collagen distribution, and extracellular matrix organization [17,18]. In this context, the present study approached AFM as an exploratory comparative tool, intended to characterize differences between mesh–tissue interfaces processed under identical experimental conditions rather than to establish absolute reference values for native tissue architecture.

The handling and pathological evaluation of explanted hernia meshes are not standardized in current clinical practice. Fadaee et al. reported a lack of consensus regarding specimen processing and the clinical value of microscopic examination and proposed gross pathological examination primarily for documentation purposes [19]. Nevertheless, histological assessment remains valuable in research settings for characterizing inflammatory infiltrates, foreign-body giant-cell reactions, fibrosis, and tissue organization. In this context, AFM may provide complementary quantitative information regarding surface-height variability, nanoscale structural heterogeneity, and fibrillar-like organization that cannot be obtained from conventional light microscopy alone. AFM should therefore be regarded as a research-oriented adjunct to, rather than a replacement for, conventional pathological assessment. In the present study, AFM was therefore used primarily as a comparative exploratory tool rather than to establish absolute reference values for native tissue architecture.

However, conventional light microscopy has a limited ability to resolve nanoscale surface topography and fine structural features of the extracellular matrix [20]. AFM provides high-resolution characterization of biological surfaces and enables assessment of surface topography and nanoscale structural organization [21]. Quantitative analysis of AFM topography can provide comparative descriptors of surface-height variability and areal surface texture under standardized experimental conditions, while computational analysis of AFM images can additionally characterize the orientation and anisotropy of elongated fibrillar features [22,23]. These measurements may complement conventional histological evaluation of tissue incorporation and pathological remodeling. Other advanced imaging approaches may provide complementary information on extracellular matrix remodeling and tissue architecture. Multiphoton techniques, particularly second harmonic generation (SHG) microscopy, enable label-free visualization of fibrillar collagen and can provide information on collagen distribution, orientation, and three-dimensional organization. Two-photon excited fluorescence and coherent Raman-based approaches, such as coherent anti-Stokes Raman scattering (CARS), can provide additional cellular or biochemical contrast, while super-resolution fluorescence microscopy can resolve selected molecular structures beyond the capabilities of conventional light microscopy [20]. These techniques interrogate different aspects of tissue organization and may therefore complement, rather than replace, AFM-based surface characterization.

Persistent extracellular matrix remodeling has previously been demonstrated in an experimental model at the interface between host tissues and polymeric materials used for hernia repair [24]. Moreover, collagen and elastin organization contribute to the anisotropic mechanical behavior of native abdominal wall structures, including the linea alba and anterior rectus sheath [25]. Alterations in fibrillar continuity and directional organization may therefore be relevant to the structural behavior of the mesh–tissue interface. In surface-roughness characterization, the measured parameters may be sensitive to scan size and the representativeness of the analyzed area [26]. Hydration state may influence AFM-derived topographical measurements [27]. More broadly, differences in sample preparation, acquisition conditions, probe–sample interactions, and data-processing procedures may affect measurement reproducibility, supporting the use of standardized protocols and identical processing of comparative specimens [28].

Previous studies have extensively described the inflammatory, fibrotic, and structural responses associated with surgical meshes [7,8,9,16,24]. However, comparatively limited information is available regarding the relationship between conventional histological findings and AFM-derived descriptors of surface organization in clinically obtained human mesh–tissue specimens. In particular, the combined use of histology and specimen-level AFM analysis to compare chronically inflamed explants with well-integrated mesh–tissue interfaces under identical processing conditions remains insufficiently explored.

The potential contribution of such a multiscale approach is not limited to confirming that chronically inflamed tissues display structural abnormalities. Rather, it may provide additional descriptors of matrix continuity, surface-height variability, and directional organization that are not routinely available through light microscopy. These descriptors may support future investigations into whether particular structural patterns are associated with unfavorable mesh incorporation, persistent symptoms, or clinically relevant implant complications. At the present stage, however, they should be regarded as exploratory morphological features rather than validated diagnostic or prognostic markers.

Accordingly, this exploratory study aimed to characterize and compare pathological and well-integrated mesh–tissue interfaces using a multiscale approach combining conventional histology and AFM. The analysis focused on inflammatory and fibrotic remodeling, collagen architecture, surface topography, areal surface parameters, and the directional organization of fibrillar-like surface features. By integrating histological observations with specimen-level AFM measurements, the study sought to evaluate whether AFM could provide complementary morphological information and establish a methodological basis for larger studies of mesh–tissue incorporation and chronic mesh-associated complications.

## 2. Materials and Methods

### 2.1. Study Design and Sample Collection

This exploratory descriptive study aimed to characterize the morphological and nanoscale surface features of the mesh–tissue interface in specimens obtained from patients with chronic mesh-associated complications. Well-integrated mesh–tissue specimens were included as a comparative morphological reference and were processed using the same histological and AFM protocols as the pathological explants.

A total of 14 mesh–tissue complexes obtained from 14 individual patients were analyzed. The pathological explant group comprised seven specimens collected during clinically indicated mesh removal. The interval between mesh implantation and explantation ranged from 6 to 9 months, with a median duration of 7 months. Five specimens were obtained following mesh removal for a foreign-body granuloma complicated by fistula formation, whereas two specimens were explanted because of hernia recurrence. Given the clinically driven nature of mesh explantation, some heterogeneity in the indication for removal was expected within the pathological group. Accordingly, these specimens were considered an exploratory pathological cohort rather than a homogeneous indication-specific population. Clinical and intraoperative findings associated with the pathological interfaces included local inflammatory changes, dense fibrosis, mesh-related tissue distortion, and fibro-inflammatory tissue adherent to the prosthetic filaments. Although mesh removal was indicated by recurrence in two patients, both specimens showed macroscopic and histological evidence of chronic fibro-inflammatory remodeling. Patient-level clinical and surgical characteristics for both study groups are summarized in Table 1.

The comparative reference group comprised seven well-integrated mesh–tissue specimens obtained during clinically indicated abdominal or pelvic surgical procedures performed for conditions unrelated to the previously implanted prosthetic material. The indications for reintervention included abdominal trauma, gynecological disorders, and digestive tract pathology. In all cases, the pre-existing mesh was not the indication for surgery.

Eligibility for inclusion in the comparative reference group required an interval of more than 12 months between mesh implantation and specimen collection, thereby reducing the potential influence of early postoperative healing and allowing the assessment of established tissue incorporation. When the mesh was directly accessible within the operative field, a small subcentimeter fragment was collected from the peripheral mesh–tissue interface. Sampling did not require removal of the entire prosthetic material and did not compromise the integrity or intended function of the remaining mesh.

Reference specimens showed no clinical or intraoperative evidence of mesh-related complications, active infection, abscess formation, tissue necrosis, marked inflammatory changes, or pronounced fibrosis-related mesh contraction. These specimens were therefore considered representative of clinically favorable mesh incorporation and were used as a comparative morphological reference rather than as healthy native-tissue controls.

All analyzed specimens contained non-absorbable synthetic prosthetic material with adherent host tissue. Where reliable clinical documentation was available, the meshes were identified as polypropylene (PP) or polyester (PET). For the remaining specimens, the exact mesh material could not be reliably retrieved from the available medical records. The commercial product and manufacturer were not consistently available; therefore, material-specific subgroup analyses were not performed.

Specimens were included only when both prosthetic material and a sufficiently preserved adherent tissue component were available for histological and AFM assessment. Specimens obtained from patients with uncontrolled systemic infection or extensive tissue necrosis that precluded reliable structural evaluation were excluded. Samples from both groups were processed and examined using identical experimental protocols.

All patients provided written informed consent for the research use of surgically obtained specimens.

### 2.2. Tissue Sampling and Processing

Specimens were collected intraoperatively during clinically indicated surgical procedures, without modifying the therapeutic objectives or overall operative strategy. In the pathological explant group, tissue fragments were selected from mesh–tissue interface regions showing macroscopic fibrotic thickening, tissue induration, and changes suggestive of chronic inflammation. In the well-integrated reference group, a peripheral subcentimeter fragment was obtained from an area showing stable incorporation of the prosthetic material into the surrounding tissue, without macroscopic evidence of active inflammation, abscess formation, tissue necrosis, or marked fibrosis-related mesh contraction. Reference sampling was performed in accordance with the approved study protocol and did not compromise the integrity, fixation, or intended function of the remaining prosthetic material.

To minimize preparation-related structural damage, specimens were excised by sharp dissection using cold instruments, and electrocautery was avoided in regions designated for histological and AFM assessment. The tissue fragments selected for analysis were prepared to an approximately uniform thickness of 0.5 mm to improve consistency of processing and comparability between specimens. This thickness was used to standardize specimen handling, mounting, and surface stability during AFM imaging; it did not represent the AFM probing depth and was not itself an analytical variable. AFM measurements were acquired from the exposed mesh–tissue interface and reflected surface topographical features.

Immediately after collection, specimens designated for AFM analysis were placed in RNAlater^®^ and transported to the laboratory. Samples were transported to the AFM laboratory within approximately 30 min after collection, and sample preparation was initiated upon arrival without prolonged storage. The overall interval between specimen collection and initiation of AFM imaging remained within approximately 1 h for all specimens. RNAlater^®^ was used solely as a standardized short-term transport and stabilization medium. Although it is primarily intended for nucleic acid preservation and is not specifically designed to preserve native extracellular matrix ultrastructure, its use provided consistent handling conditions and limited the interval between specimen excision and AFM preparation. The same collection, transport, stabilization, rinsing, and subsequent AFM preparation protocol was applied to both study groups to minimize preparation-related variability and ensure comparability between specimens.

Before mounting and AFM imaging, specimens were thoroughly rinsed with Dulbecco’s phosphate-buffered saline (DPBS; 1×, pH 7.0, Thermo Fisher Scientific, Waltham, MA, USA) to remove residual RNAlater^®^ (Thermo Fisher Scientific, Austin, TX, USA) and standardize the preparation conditions. Consequently, AFM findings were interpreted as comparative morphological observations obtained under the standardized protocol used in this study rather than as absolute representations of native, fully hydrated extracellular matrix ultrastructure.

### 2.3. Histological Analysis

Tissue fragments designated for histological examination were fixed in 10% neutral-buffered formalin, processed according to standard histopathological procedures, embedded in paraffin, and sectioned at a thickness of 4 µm.

Sections were stained with hematoxylin and eosin (H&E) to describe overall tissue architecture, cellularity, inflammatory infiltrates, macrophages, foreign body giant-cell reaction, vascular structures, and other morphological features associated with chronic inflammation. Van Gieson staining was used to describe collagen deposition, collagen bundle arrangement, fibrosis, and extracellular matrix organization at the mesh–tissue interface.

All available histological sections were evaluated qualitatively by a specialist in anatomic pathology, and representative microscopic fields were selected from each specimen. In the pathological explant group, the assessment focused on chronic inflammatory infiltrates, foreign body-associated tissue reaction, fibro-inflammatory remodeling, fibrosis, and alterations in collagen continuity, distribution, and orientation. In the well-integrated reference group, the evaluation focused on fibrous incorporation around the prosthetic material, collagen continuity and arrangement, and the presence and spatial distribution of the associated inflammatory response.

Histological findings were interpreted as conventional morphological descriptors and were used to contextualize the AFM-derived observations of surface topography, fibrillar-like organization, and directionality. Because AFM characterizes surface morphology rather than tissue composition directly, fibrillar-like surface features were interpreted in relation to the collagen-rich architecture observed histologically and were not identified as collagen fibers solely on the basis of AFM imaging.

No formal histological scoring system, quantitative morphometric analysis, or collagen-specific polarization analysis was performed. Accordingly, histological comparisons between the two groups were descriptive and were not intended to provide quantitative estimates of inflammatory burden or collagen organization. Histological assessment was therefore used to provide qualitative morphological context for the quantitative AFM analysis rather than as an independent quantitative histopathological endpoint.

Both H&E- and Van Gieson-stained sections were included in the histological assessment. Representative Van Gieson-stained sections were selected for the comparative figures because this staining method provided clearer visualization of collagen distribution and extracellular matrix organization at the mesh–tissue interface.

### 2.4. AFM Sample Preparation

For AFM analysis, tissue fragments from both the pathological explant group and the well-integrated reference group were mounted on poly-L-lysine-coated glass slides to provide adequate adhesion and mechanical stability during image acquisition.

Before mounting, specimens were gently rinsed with Dulbecco’s phosphate-buffered saline (DPBS; 1×, pH 7.0) to remove residual transport medium and loosely adherent surface material. Excess buffer was carefully removed without compressing or mechanically deforming the tissue. The specimens were then positioned with the mesh–tissue interface exposed and allowed to adhere to the coated glass substrate.

AFM measurements were performed in air at room temperature, approximately 22–24 °C, while the specimens remained partially hydrated. Because imaging in air may allow progressive moisture loss, AFM-derived measurements were interpreted comparatively between specimens processed under the same conditions rather than as absolute native-tissue values. Following rinsing, trimming as required, and mounting, AFM imaging was initiated as soon as specimen preparation was completed. At the start of imaging, the specimens remained partially hydrated and had not undergone complete surface dehydration.

The partially hydrated state was selected as part of the exploratory protocol to facilitate specimen immobilization while avoiding complete dehydration before imaging. However, imaging in air may permit progressive moisture loss and consequently influence tissue dimensions and AFM-derived surface parameters. Because the objective of the study was comparative rather than to establish absolute native-tissue topographical values, the same preparation, hydration, mounting, and imaging procedure was applied to all specimens from both groups. Accordingly, AFM-derived measurements were interpreted as standardized comparative morphological descriptors obtained under the experimental conditions of this study rather than as absolute measurements of native, fully hydrated tissue ultrastructure.

### 2.5. AFM Imaging and Morphological Analysis

Nanoscale surface characterization was performed using an EasyScan 2 atomic force microscope (Nanosurf AG, Liestal, Switzerland) equipped with the manufacturer’s acquisition and analysis software. The same acquisition and data-processing protocol was applied to specimens from both the pathological explant group and the well-integrated reference group.

AFM imaging was performed in contact mode using PPP–NCSTAuD probes (Nanosensors, Neuchâtel, Switzerland) with a conical tip, a nominal spring constant of approximately 0.4 N/m, and a nominal tip radius of less than 7 nm. The probes were commercially manufactured and used as supplied, without chemical or mechanical tip fabrication or modification by the authors. A force setpoint of 12 nN was used, corresponding to the minimum force required to maintain stable tip–sample contact, and images were acquired at a scan rate of 0.6 Hz. Contact mode was selected as part of the exploratory protocol because it provided stable surface tracking of tissue fragments immobilized on poly-L-lysine-coated glass slides. All specimens were examined in a partially hydrated state under comparable environmental and instrumental conditions.

Because continuous tip–sample contact may deform or displace soft biological structures, potential interaction artifacts were assessed by monitoring image stability and inspecting the acquired images for streaking, dragging, discontinuities, tissue displacement, folds, detached material, edge effects, or other features suggestive of excessive interaction forces or unstable feedback. Regions showing such artifacts were excluded from analysis. Artifact assessment was not performed blinded to specimen group allocation. Specimen immobilization, environmental conditions, and scanning procedures were kept consistent across all samples to reduce acquisition-related variability.

Topographical images were acquired over standardized areas of 2 × 2 µm^2^. A total of 64 scans were analyzed per specimen as a technical sampling strategy across multiple regions of interest to capture local surface heterogeneity; these scans were not intended to provide exhaustive mapping of the entire mesh–tissue interface. Only images showing stable tip–sample interaction and clearly resolved surface morphology were retained. Repeated scans obtained from the same specimen were considered technical subsamples rather than independent biological replicates.

Raw topographical datasets were processed using least-squares plane leveling to correct specimen tilt and global surface inclination. A Gaussian L-filter with a cutoff wavelength of 0.8 µm was subsequently applied to reduce long-wavelength components associated with residual surface form and to facilitate assessment of local micro- and nanoscale surface features.

No S-filter was applied because the analysis aimed to preserve fine-scale features potentially contributing to fibrillar-like surface organization. The absence of an S-filter may also retain high-frequency noise; therefore, datasets showing evident noise-related or acquisition artifacts were excluded following visual quality assessment. The instrumental noise floor was not independently quantified in the present study; therefore, residual high-frequency instrumental noise cannot be completely excluded. The same leveling and filtering workflow was applied to all retained datasets.

Because AFM-derived surface parameters may be influenced by specimen hydration, contact-mode imaging, probe–sample interaction, and data-processing settings, the measurements were interpreted as standardized comparative morphological descriptors obtained under the experimental conditions of this study. They were not considered absolute measurements of native tissue topography or validated diagnostic thresholds.

### 2.6. Areal Surface Parameter Analysis

Areal surface texture parameters were calculated from the processed AFM topography datasets according to the definitions provided in ISO 25178-2 [22]. The evaluated parameters included Sa (arithmetic mean height), Sq (root mean square height), Ssk (skewness of the height distribution), Sku (kurtosis of the height distribution), Sp (maximum peak height), Sv (maximum pit depth), and Sz (maximum surface height).

These parameters were used to describe surface-height variability, the shape and symmetry of the height distribution, the presence of extreme topographical features, and the overall heterogeneity of the mesh–tissue interface. Sa and Sq were used as measures of average surface amplitude, whereas Ssk and Sku characterized the distribution of surface heights. Sp, Sv, and Sz described the vertical extent of prominent peaks, depressions, and the overall peak-to-valley range. In pathological explants, these parameters were used to characterize surface irregularity in the context of chronic fibro-inflammatory remodeling.

Because AFM-derived roughness values may be influenced by tissue hydration, specimen preparation, contact-mode imaging, probe–sample interaction, and data-filtering settings, they were interpreted as descriptive parameters obtained under the standardized experimental conditions of this study. They were not considered standalone diagnostic indicators or absolute measurements of native tissue surface architecture. No correction for multiple comparisons was applied because of the exploratory nature of the analysis; therefore, the resulting *p*-values should be interpreted accordingly.

### 2.7. Directionality Analysis

The spatial organization of surface features was evaluated using directional analysis of the processed AFM topography datasets. This analysis was used to examine the distribution of local orientations among elongated fibrillar-like and ridge-like surface features and to identify the presence or absence of preferential alignment at the mesh–tissue interface.

Directional analysis was performed using the same image-processing workflow for specimens from both the pathological explant group and the well-integrated reference group. Representative outputs were selected to illustrate the morphological patterns observed in each group; no specimen-level inferential statistical comparison of directional parameters was performed.

Because AFM topography reflects surface geometry rather than the histological composition of individual structures, fibrillar-like features were not identified as collagen fibers solely on the basis of AFM imaging. Their organization was interpreted in conjunction with the corresponding histological findings. Directionality was therefore considered a complementary morphological descriptor and not a standalone diagnostic or prognostic criterion.

### 2.8. Data Analysis

Data analysis followed an exploratory comparative approach integrating qualitative histological assessment with specimen-level quantitative AFM analysis. Histological findings were evaluated descriptively, with emphasis on chronic inflammatory infiltrates, foreign body giant-cell reaction, fibro-inflammatory remodeling, fibrosis, collagen continuity and organization, and overall extracellular matrix architecture. Directional findings were also interpreted descriptively as complementary morphological features.

Sixty-four AFM scans were analyzed per specimen as a technical sampling strategy across multiple regions of interest to capture local surface heterogeneity; these scans were not intended to provide exhaustive mapping of the entire mesh–tissue interface. For each areal surface parameter—Sa, Sq, Ssk, Sku, Sp, Sv, and Sz—the scan-level measurements were averaged within each specimen, yielding one specimen-level value. Accordingly, seven independent values were available for the pathological explant group and seven for the well-integrated reference group. Repeated scans obtained from the same specimen were treated as technical subsamples and were not considered independent biological replicates.

Given the limited number of biological specimens and the exploratory design, between-group comparisons of AFM surface parameters were performed using the exact two-sided Mann–Whitney U test. Results were summarized as median and interquartile range. The Mann–Whitney U statistic was reported as the smaller of the two group-specific U values, and exact two-sided *p*-values were reported to six decimal places. A two-sided *p*-value below 0.05 was considered statistically significant.

No inferential statistical testing was applied to the qualitative histological findings or to the representative directional analysis. Statistical differences in AFM-derived surface parameters were interpreted as exploratory group-level findings and were not used to establish diagnostic, prognostic, or classification thresholds.

## 3. Results

### 3.1. Histological Findings

**Figure 1:**
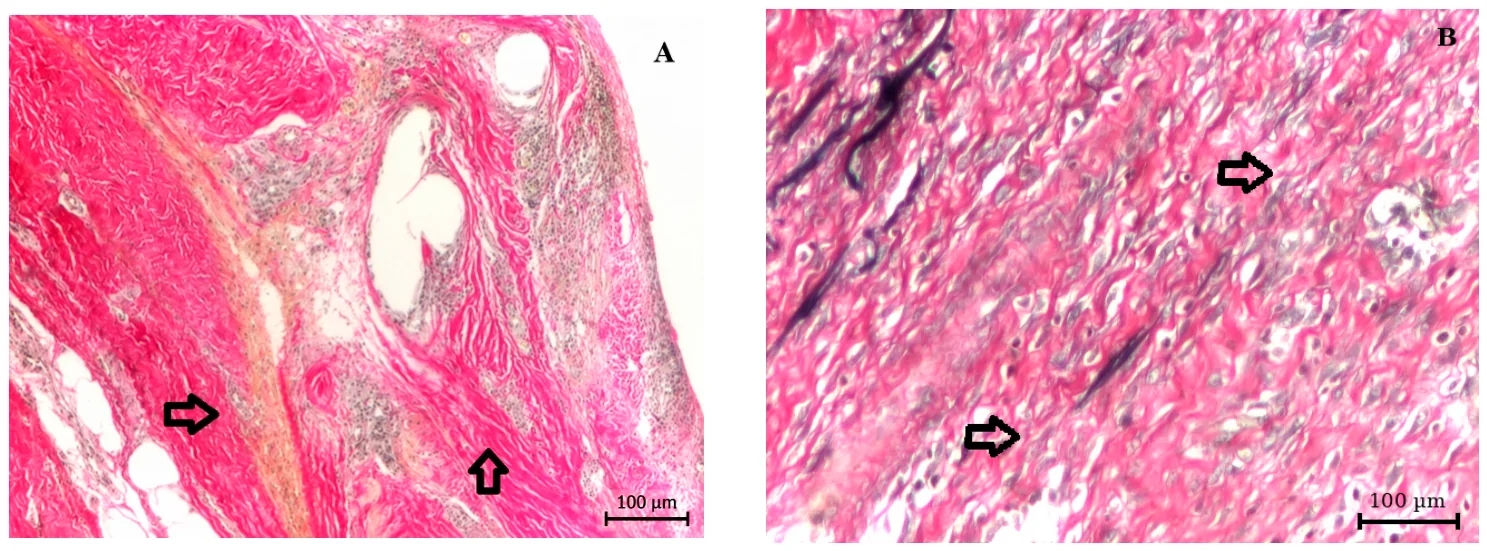
Comparative histological appearance of the mesh–tissue interface. (**A**) Well-integrated reference specimen showing relatively compact and continuous collagen bundles surrounding mesh-associated spaces, with focal inflammatory cellularity. Arrows indicate representative areas of relatively continuous collagen organization. (**B**) Pathological explant showing prominent fibro-inflammatory tissue, inflammatory cellularity, and irregular collagen bundles with variable thickness, discontinuous organization, and non-uniform orientation. Arrows indicate representative areas of disrupted and irregular collagen organization. Van Gieson staining; bright-field light microscopy; original magnification, ×10; scale bars, 100 µm.

Qualitative examination of H&E- and Van Gieson-stained sections showed differences in inflammatory cellularity and extracellular matrix organization between the two study groups. Representative Van Gieson-stained sections are presented in Figure 1 to illustrate the comparative collagen architecture at the mesh–tissue interface.

Well-integrated reference specimens showed relatively compact and continuous collagen-rich tissue surrounding mesh-associated spaces, with a comparatively coherent arrangement of collagen bundles and only focal inflammatory cellularity (Figure 1A). In contrast, pathological explants showed more prominent fibro-inflammatory tissue and inflammatory cellularity, with collagen bundles of variable thickness, less continuity, and less uniform orientation (Figure 1B).

Overall, the histological findings suggested comparatively preserved collagen organization in the well-integrated reference specimens and greater fibro-inflammatory heterogeneity and extracellular matrix disorganization in the pathological explants. Because no formal histological scoring or quantitative morphometric analysis was performed, these observations were interpreted descriptively.

### 3.2. AFM Deflection Imaging

**Figure 2:**
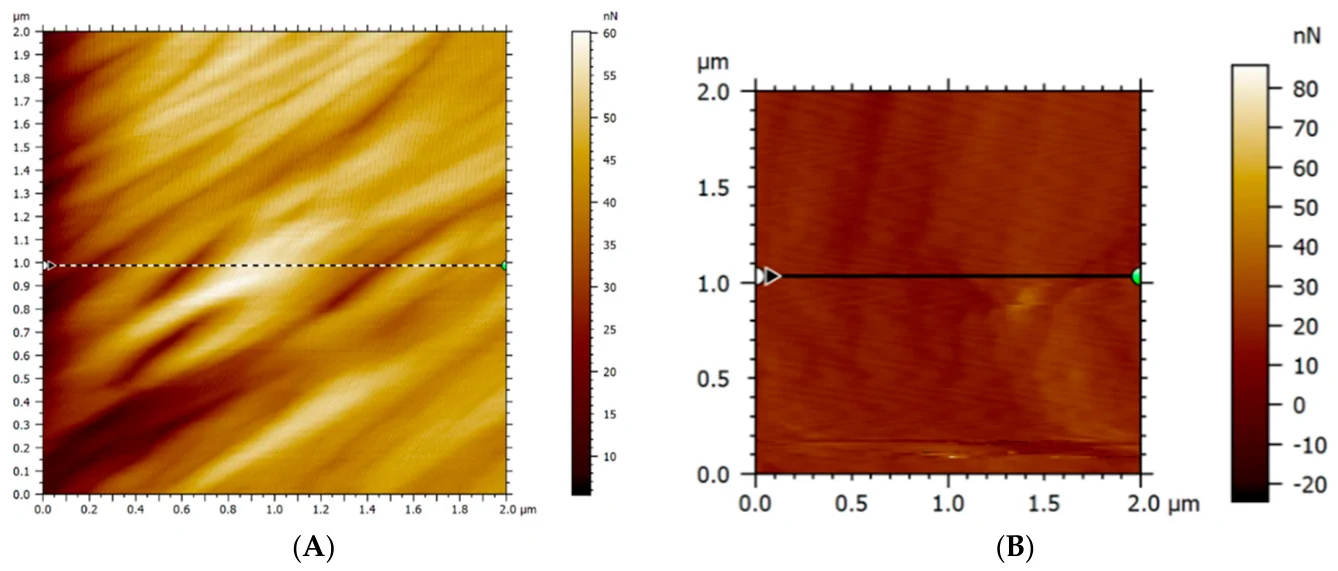
Comparative AFM deflection maps of the mesh–tissue interface. (**A**) Well-integrated reference specimen showing relatively continuous and partially oriented surface features. (**B**) Pathological explant showing greater signal heterogeneity, irregular structural distribution, and reduced continuity. Contact-mode AFM; identical lateral spatial scale in both panels; scan area, 2 × 2 µm^2^; specimens examined in a partially hydrated state. Deflection display ranges were independently scaled to preserve morphological detail in each representative image.

AFM deflection maps showed different surface-signal patterns in representative mesh–tissue interfaces from the two study groups. The well-integrated reference specimen displayed elongated and relatively continuous surface features, with evidence of partial preferential orientation and a comparatively uniform distribution of the contact-mode deflection signal (Figure 2A).

In contrast, the pathological explant showed greater submicron-scale signal heterogeneity, with irregular, intersecting, and discontinuous surface features and a less uniform distribution of the deflection signal (Figure 2B). When considered together with the histological findings of fibro-inflammatory remodeling and altered collagen organization, these observations were consistent with greater surface disorganization in the pathological interface.

### 3.3. AFM Topography

**Figure 3:**
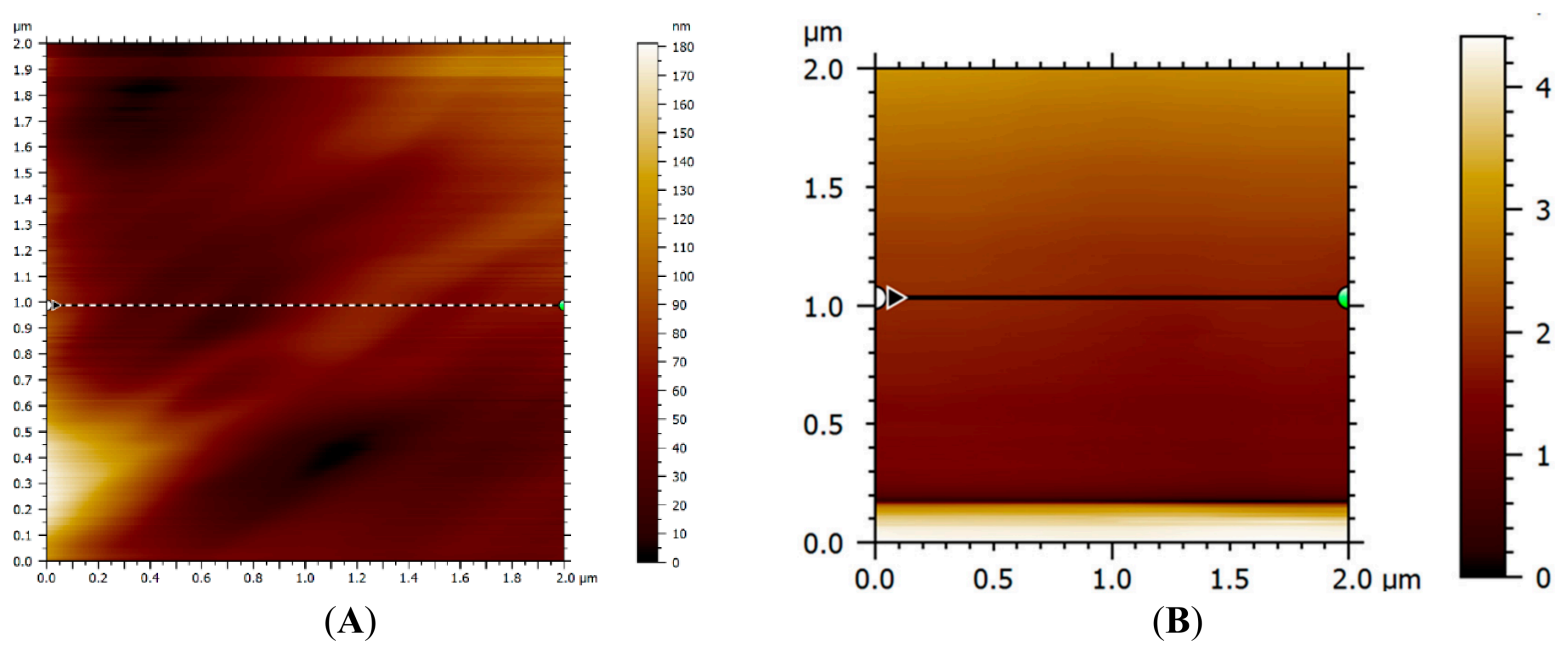
Comparative AFM topography of the mesh–tissue interface. (**A**) Well-integrated reference specimen showing a heterogeneous but relatively continuous surface with partially oriented elongated features. (**B**) Pathological explant showing greater topographical heterogeneity, irregular elevations and depressions, and reduced structural continuity.

AFM topography revealed different surface-morphology patterns in representative specimens from the two study groups. The well-integrated reference specimen displayed a heterogeneous but comparatively continuous surface, with height variations of approximately 0–180 nm across the 2 × 2 µm^2^ scanning area. Elongated ridge-like features showed a comparatively consistent oblique orientation, suggesting a more organized fibrillar-like surface pattern at the mesh–tissue interface (Figure 3A).

In contrast, the representative pathological explant exhibited greater topographical heterogeneity, with irregularly distributed elevations and depressions, discontinuous fibrillar-like features, and reduced surface continuity (Figure 3B). A linear feature visible along the lower margin of the pathological image was considered a scanning- or boundary-related artifact and was excluded from quantitative analysis.

When considered together with the histological findings, the comparatively continuous and partially oriented surface pattern observed in the well-integrated specimen was consistent with more favorable tissue incorporation. Conversely, the irregular and discontinuous topography of the pathological explant was compatible with the fibro-inflammatory remodeling and altered extracellular matrix organization observed histologically.

Contact-mode AFM; identical lateral spatial scale in both panels; scan area, 2 × 2 µm^2^. Height display ranges were independently scaled to preserve morphological detail in each representative image.

### 3.4. Three-Dimensional AFM Reconstruction

**Figure 4:**
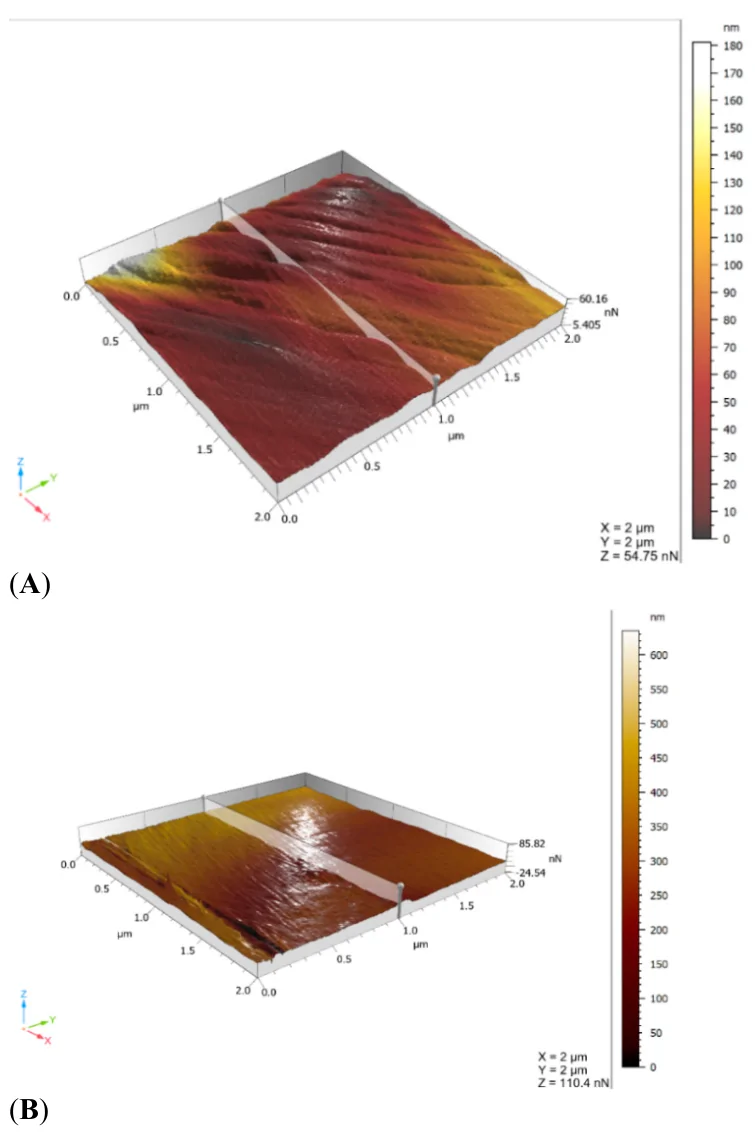
Comparative three-dimensional AFM topography of the mesh–tissue interface. (**A**) Well-integrated reference specimen showing comparatively continuous relief, gradual height transitions, and partially oriented elongated surface features. (**B**) Pathological explant showing irregular elevations and depressions, steeper gradients, and greater structural discontinuity. Contact-mode AFM; scan area, 2 × 2 µm^2^. Vertical display ranges were independently scaled to preserve morphological detail in each representative surface; quantitative between-group comparisons are based on specimen-level AFM parameters reported in Table 2.

Three-dimensional AFM reconstruction further illustrated differences in surface morphology between representative specimens from the two study groups. The well-integrated reference specimen showed a heterogeneous but comparatively continuous surface relief, characterized by elongated ridge-like features, gradual height transitions, and a partially preferential orientation across the scanned area (Figure 4A).

In contrast, the representative pathological explant displayed a more irregular surface relief, with prominent elevations and depressions, steeper local height transitions, and discontinuous surface domains lacking a consistent spatial arrangement (Figure 4B).

When considered together with the histological findings, the comparatively continuous three-dimensional topography of the well-integrated specimen was consistent with more organized tissue incorporation. Conversely, the fragmented and heterogeneous surface morphology of the pathological explant was compatible with the fibro-inflammatory remodeling and altered extracellular matrix organization observed histologically.

### 3.5. Specimen-Level Comparison of Areal Surface Parameters

Areal surface parameters were calculated from the processed AFM topography datasets according to ISO 25178-2. For each specimen, the values obtained from 64 scans were averaged to generate one specimen-level value for each parameter. The resulting seven independent values per group were summarized as median and interquartile range and compared using the exact two-sided Mann–Whitney U test. The same least-squares plane-leveling procedure and Gaussian L-filter with a cutoff wavelength of 0.8 µm, without an S-filter, were applied to all datasets.

The pathological explant group showed higher Sa, Sq, Sku, Sp, Sv, and Sz values than the well-integrated reference group (Table 2). Median Sa was 6.655 nm in the well-integrated group and 13.060 nm in the pathological group, while median Sq was 8.387 and 27.930 nm, respectively. Larger differences were observed for the extreme-height parameters: median Sp was 44.20 versus 304.10 nm, median Sv was 22.90 versus 285.30 nm, and median Sz was 67.13 versus 590.30 nm in the well-integrated and pathological groups, respectively.

The pathological group also showed substantially higher Sku values, with a median of 31.820 compared with 4.038 in the well-integrated group, consistent with a height distribution containing more pronounced extreme topographical features. In contrast, Ssk was positive in the well-integrated group and negative in the pathological group, indicating different asymmetries in the surface-height distributions. All between-group differences were statistically significant in this dataset, with U = 0 and an exact two-sided *p*-value of 0.000583 for each parameter. In practical terms, U = 0 indicated complete rank separation between the two groups for each parameter in this dataset: all pathological specimen-level values were higher than the corresponding well-integrated values for Sa, Sq, Sku, Sp, Sv, and Sz, whereas all pathological Ssk values were lower. Given the small sample size, this complete separation should be interpreted as a feature of the present dataset rather than as evidence of universally applicable thresholds.

### 3.6. Representative Surface Profiles

**Figure 5:**
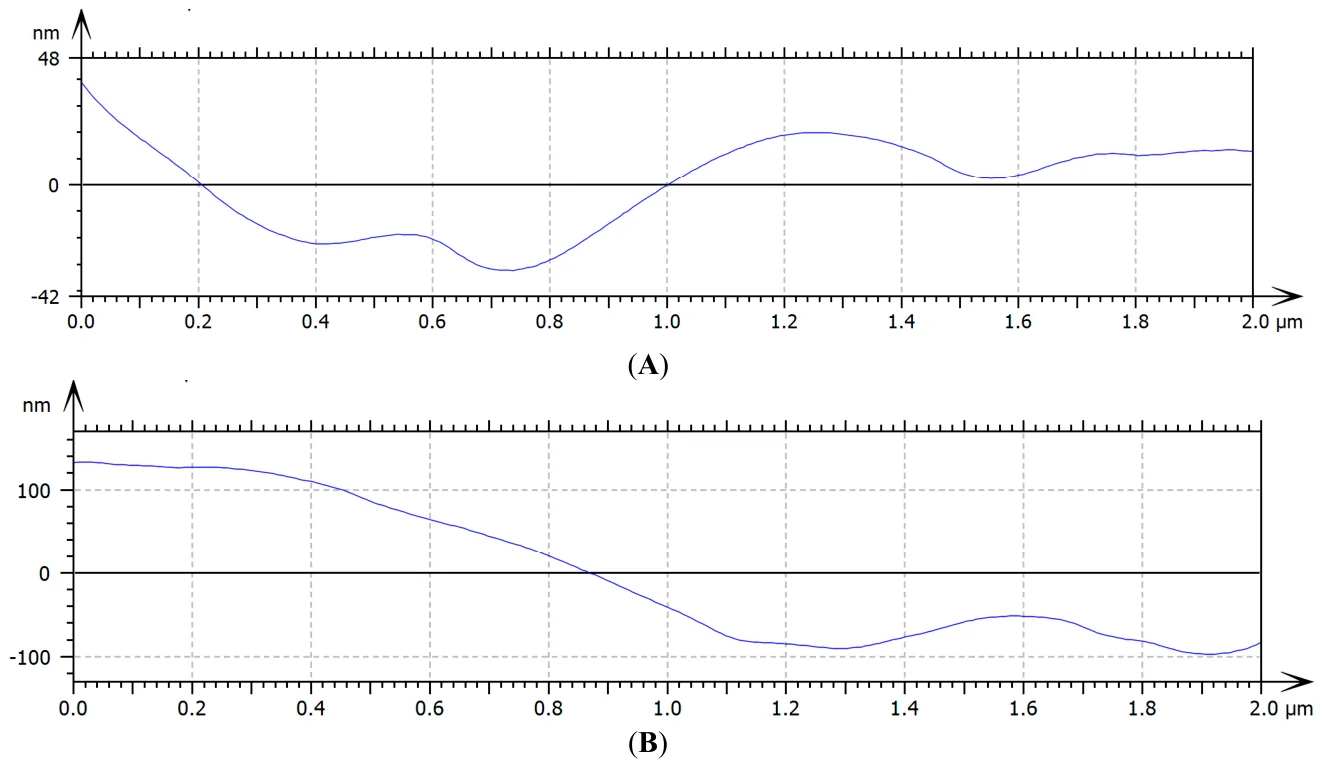
Surface profiles extracted from representative AFM topography scans of the mesh–tissue interface. (**A**) Well-integrated reference scan showing gradual and relatively continuous height variations. (**B**) Pathological scan showing a steeper height gradient, abrupt transitions, and irregular local oscillations. Profile length: approximately 2 µm.

Surface profiles extracted from the central region of representative AFM topography scans illustrated different patterns of height variation in the two study groups. The well-integrated reference scan showed gradual and comparatively continuous nanometric height variations, without pronounced abrupt transitions, consistent with a more uniform surface relief (Figure 5A).

In contrast, the representative pathological scan displayed a steep height variation exceeding 200 nm over approximately the first 1 µm of the profile, followed by an abrupt transition and irregular local oscillations of approximately 30–60 nm (Figure 5B). This pattern illustrated greater local topographical heterogeneity and surface discontinuity and was consistent with the higher specimen-level surface-amplitude and peak-to-valley parameters observed in the pathological group.

### 3.7. Directionality and Structural Organization

**Figure 6:**
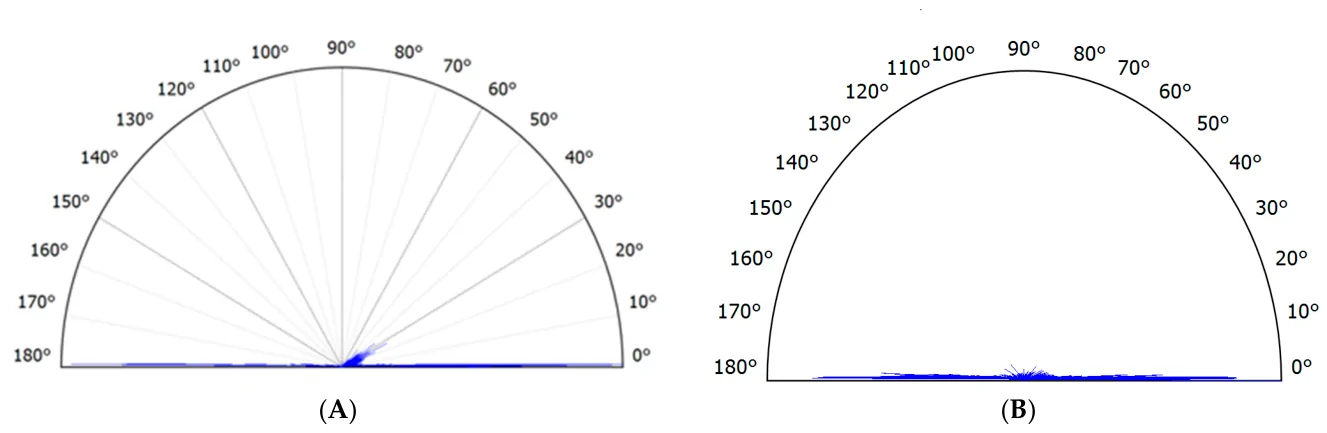
Comparative directional distribution of surface features derived from representative AFM topography scans. (**A**) Well-integrated interface showing a more concentrated angular distribution and a comparatively clearer preferential orientation. (**B**) Pathological interface showing a broader and less clearly defined directional distribution, consistent with reduced structural organization. Contact-mode AFM; scan area, 2 × 2 µm^2^.

The directional distribution of surface-feature orientations derived from the processed AFM topography datasets was visualized using polar histograms (Figure 6). This analysis characterized the local orientation of elongated ridge-like and fibrillar-like surface features and was used to assess whether their angular distribution showed preferential alignment or a broader, less organized pattern.

The representative well-integrated interface showed a more concentrated angular distribution, with a comparatively clearer preferential orientation (Figure 6A). In contrast, the representative pathological interface displayed a broader and less clearly defined distribution of orientations, suggesting reduced directional organization of the surface pattern (Figure 6B).

Importantly, the directional analysis was derived exclusively from the AFM topography channel rather than from the deflection signal. It therefore describes the geometric organization of surface features and should not be interpreted as a direct measurement of local mechanical properties.

**Figure 7:**
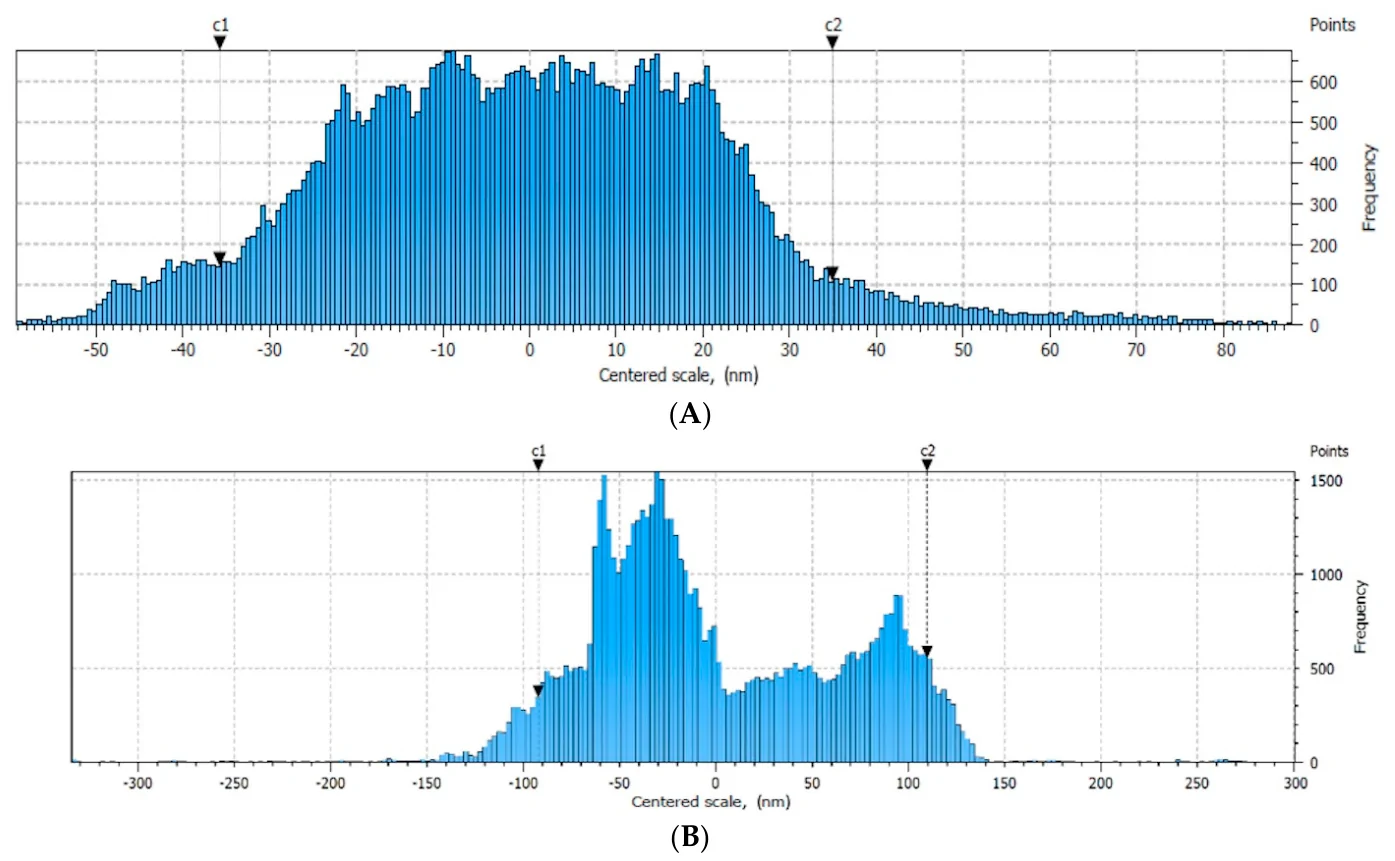
Representative surface-height distributions derived from AFM topography scans of the mesh–tissue interface. (**A**) Well-integrated reference interface showing a comparatively narrower and more compact height distribution. (**B**) Pathological interface showing a broader, more irregular, and asymmetric height distribution, consistent with greater topographical heterogeneity. Contact-mode AFM; scan area, 2 × 2 µm^2^.

Additional information regarding the distribution of surface heights is presented in Figure 7. The representative well-integrated interface showed a comparatively narrower and more compact height distribution (Figure 7A), whereas the pathological interface displayed a broader, more irregular and asymmetric distribution (Figure 7B), consistent with greater topographical heterogeneity.

The representative pathological interface exhibited a broad asymmetric profile, suggesting the coexistence of elevated regions and pronounced depressions. In contrast, the representative well-integrated interface showed a relatively more compact distribution of surface heights, indicative of a more uniform topographical organization.

## 4. Discussion

Chronic mesh-associated complications reflect a complex interaction between the implanted material, the host foreign body response, extracellular matrix remodeling, and the mechanical environment of the abdominal wall [7,9,16,24,25]. In this exploratory study, conventional histology and AFM were combined to compare pathological mesh–tissue explants with clinically well-integrated reference specimens processed under identical experimental conditions. The pathological interfaces showed more pronounced fibro-inflammatory remodeling, reduced collagen continuity, and greater surface heterogeneity, whereas the well-integrated interfaces retained a comparatively more continuous and organized collagen-rich architecture. Specimen-level AFM analysis further demonstrated significant differences in all evaluated areal surface parameters, supporting and extending the qualitative histological observations. The observed histological and AFM differences are likely multifactorial, potentially reflecting the combined effects of persistent foreign-body inflammation, fibro-inflammatory extracellular matrix remodeling, implantation duration, local mechanical conditions, mesh characteristics, and the clinical processes leading to reoperation.

The medico-surgical context of the pathological specimens is relevant to the interpretation of these findings. Five of the seven meshes were removed because of foreign-body granuloma complicated by fistula formation, whereas two were explanted in the setting of hernia recurrence. The implantation interval ranged from 6 to 9 months, with a median duration of 7 months. These indications represent clinically distinct patterns of unfavorable postoperative evolution. Fistula formation is consistent with a persistent local pathological process requiring operative management, whereas recurrence may result from insufficient durable reinforcement, unfavorable tissue incorporation, technical factors, patient-related vulnerability, or interactions among these mechanisms [1,2,9]. The present findings do not establish the specific cause of recurrence and do not demonstrate that the AFM patterns directly produced the clinical complications. Given the small and unequal subgroups, no indication-specific AFM analysis was performed. Therefore, the present study cannot determine whether fistulizing foreign-body granuloma and hernia recurrence are associated with distinct AFM patterns.

Histological examination demonstrated a foreign body-associated tissue response in both groups. This finding is expected because implantation of a synthetic mesh normally induces macrophage recruitment, multinucleated giant-cell formation, fibroblast activation, fibrous tissue deposition, and remodeling of the surrounding extracellular matrix [7,16,24]. The presence of inflammatory cells or a foreign body response alone should therefore not be considered evidence of pathological mesh failure. Rather, the groups differed primarily in the distribution and structural organization of the tissue response. Well-integrated specimens showed relatively compact and continuous collagen-rich connective tissue with focal inflammatory cellularity, whereas pathological explants showed more prominent fibro-inflammatory tissue and inflammatory cellularity, irregular collagen deposition, and reduced continuity of collagen bundles. These observations are compatible with previously described patterns of persistent foreign body response and pathological remodeling at the interface with implanted polymers [16,24].

From a surgical perspective, favorable mesh incorporation requires more than the development of fibrosis around prosthetic filaments. The newly formed tissue should provide stable structural continuity between the implant and the surrounding abdominal wall without excessive contraction, persistent inflammation, infection, fistula formation, or distortion of the reconstructed anatomy [1,2,9]. Dense fibrosis may therefore reflect either effective incorporation or an unfavorable tissue response, depending on its distribution, organization, relationship with the prosthetic material, and clinical consequences. In the pathological specimens examined in the present study, fibrosis was associated with increased structural heterogeneity, irregular collagen arrangement, and reduced continuity of collagen bundles. These findings suggest that the quantity of fibrous tissue alone may not adequately characterize the quality of mesh incorporation.

AFM provided complementary information regarding the surface morphology of the mesh–tissue interface [21]. Representative well-integrated scans showed comparatively continuous ridge-like features, gradual height transitions, and a more clearly defined preferential orientation. In contrast, pathological scans displayed irregular elevations and depressions, discontinuous surface features, and greater local heterogeneity. When interpreted together with the histological findings, these observations were consistent with less organized tissue remodeling at pathological interfaces. However, AFM topography primarily characterizes surface geometry rather than molecular composition. Ridge-like or fibrillar-like features therefore cannot be identified as collagen fibers solely on the basis of AFM imaging, and AFM does not independently establish the cellular or molecular mechanisms responsible for the observed morphology [21].

The specimen-level quantitative results supported these morphological differences. Measurements from 64 scans per specimen were averaged to obtain one value for each of the seven independent biological specimens in each group. Median Sa was 13.060 nm in the pathological group compared with 6.655 nm in the well-integrated group, while median Sq was 27.930 versus 8.387 nm. More pronounced differences were observed for the extreme-height parameters: median Sp was 304.10 versus 44.20 nm, median Sv was 285.30 versus 22.90 nm, and median Sz was 590.30 versus 67.13 nm in the pathological and well-integrated groups, respectively. All between-group differences were statistically significant in the present dataset, with U = 0 and an exact two-sided *p*-value of 0.000583 for each parameter.

Higher Sa and Sq values indicated greater average surface-height variability in the pathological interfaces, whereas the higher Sp, Sv, and Sz values reflected more prominent elevations, deeper depressions, and a substantially broader peak-to-valley range. Median Sku was also markedly higher in the pathological group, at 31.820 compared with 4.038 in the well-integrated group, indicating a height distribution containing more pronounced extreme topographical features. In addition, Ssk was negative in the pathological group and positive in the well-integrated group, reflecting different asymmetries of the surface-height distributions. Taken together, these parameters suggest that pathological interfaces were not merely rougher on average but also contained more irregular and extreme surface features.

These quantitative differences strengthen the morphological comparison between the groups, but they should be interpreted cautiously. The complete numerical separation observed in the present dataset does not indicate that the reported values represent diagnostic thresholds or universally applicable reference ranges. Instead, the parameters should be regarded as exploratory group-level descriptors obtained under the standardized preparation, imaging, and processing conditions used in this study. More generally, scan size and the representativeness of the analyzed surface area may substantially influence surface-roughness characterization [26]. Hydration state may represent an additional source of variability in AFM-derived topographical measurements [27]. More broadly, differences in sample preparation, acquisition conditions, probe–sample interaction, and data-processing procedures may affect measurement reproducibility, supporting the use of standardized protocols [28]. A potential influence of RNAlater^®^ on the absolute AFM measurements cannot be excluded because untreated paired specimens were not available for comparison. However, all specimens underwent the same short-term RNAlater^®^ exposure, transport interval, rinsing procedure, mounting, and AFM acquisition protocol. Therefore, the present findings should be interpreted as comparative differences obtained under standardized experimental conditions rather than as absolute representations of native tissue ultrastructure.

Directional analysis provided additional descriptive information regarding the spatial organization of elongated surface features. The representative well-integrated interface showed a more concentrated angular distribution and a comparatively clearer preferential orientation, whereas the pathological interface displayed a broader and less clearly defined directional pattern. These findings suggested reduced directional consistency of surface organization in the pathological specimen. Nevertheless, the analysis was based on representative AFM topography images and was not compared statistically at specimen level. It therefore does not demonstrate quantitative group-level differences in collagen-fiber alignment, mechanical anisotropy, or directional load transmission. The anisotropic mechanical behavior of native abdominal wall structures depends partly on the organization of collagen and elastin [25], while collagen fibrils themselves display complex time-dependent and inelastic mechanical behavior [29]. The present morphological analysis did not directly measure these mechanical properties.

The directional findings may nevertheless be relevant within a broader mechanobiological context. Extracellular matrix alignment can influence focal-adhesion organization and directional cell migration [30]. During wound healing, extracellular matrix deposition, degradation, and reorganization are dynamically coordinated processes that may generate structurally heterogeneous tissue domains [31]. In the presence of persistent inflammation and an implanted polymer, these remodeling processes may continue at the tissue–material interface [11,24]. Cellular force transmission also depends on the organization of cytoskeletal stress fibers and their interactions with adhesion structures [32]. However, these cellular and mechanical processes were not evaluated directly in the present study. Consequently, the AFM findings should not be interpreted as evidence of altered cell migration, mechanotransduction, or tissue-level mechanical competence.

Taken together, the histological and AFM findings support the interpretation that pathological mesh–tissue interfaces are characterized by a less continuous and more heterogeneous extracellular matrix organization. In the present study, irregular collagen arrangement on histological examination was observed alongside greater surface-height variability and more pronounced extreme topographical features on AFM. The two methods therefore provided complementary information at different spatial scales: histology demonstrated the cellular and collagen-rich tissue response, whereas AFM characterized the surface geometry and nanoscale heterogeneity of the interface. Nevertheless, because the histological assessment was qualitative, direct quantitative correlations between histological changes and individual AFM parameters could not be established.

The combined findings may have relevance to the interpretation of chronic mesh complications encountered in surgical practice. A pathological mesh–tissue interface is unlikely to be defined by a single morphological feature. Symptoms and intraoperative findings may result from interactions among persistent inflammation, excessive or irregular fibrosis, mesh contraction, infection, fistula formation, recurrence, fixation-related factors, and altered abdominal wall mechanics [1,2,9,15,16,25]. The present results suggest that unfavorable interfaces may also display measurable differences in nanoscale surface organization. However, it remains uncertain whether these differences preceded the clinical manifestations, developed secondary to persistent inflammation or mechanical disturbance, or resulted from interactions between these processes.

The observed AFM differences should not currently influence clinical decisions regarding preservation or explantation of an implanted mesh. Mesh removal remains an individualized medico-surgical decision based on the clinical indication for explantation, the extent and location of the mesh-related complication, mesh characteristics, operative feasibility, patient physiology, and the implications for subsequent abdominal wall reconstruction [5,33]. AFM requires excised tissue, specialized equipment, and controlled sample preparation and therefore has no immediate role as a routine preoperative or intraoperative diagnostic method. Its more realistic potential lies in research-oriented characterization of explanted materials and in comparative studies examining how mesh type, implantation plane, fixation technique, contamination, implantation duration, and clinical indication relate to tissue incorporation.

The multiscale approach may also contribute to future biomaterial research. Previous multiscale studies have similarly demonstrated the value of integrating microstructural characterization with mechanical analysis to elucidate structure–property relationships in biological tissues and biomaterials [34,35]. Compared with these advanced optical approaches, AFM offers nanometer-scale topographical characterization and direct quantitative assessment of local surface morphology, but it examines relatively small fields of view and is sensitive to specimen preparation, hydration state, and tip–sample interactions. SHG and multiphoton imaging can interrogate larger tissue regions and provide three-dimensional information on collagen architecture with minimal or no exogenous labeling, but they do not provide the same direct surface-height measurements as AFM. Coherent Raman and super-resolution approaches can add biochemical or molecularly specific information, although they require specialized instrumentation and, depending on the technique, more complex acquisition, labeling, and data-analysis workflows [20]. These complementary strengths support the use of multimodal strategies for investigating mesh integration and extracellular matrix remodeling rather than reliance on a single imaging modality. Histology characterizes inflammatory cellularity, fibrosis, foreign body giant-cell reaction, and collagen organization, whereas AFM provides complementary descriptors of surface-height variability, extreme topographical features, directional organization, and local structural continuity. Integrating these observations may help distinguish dense but organized tissue incorporation from heterogeneous fibro-inflammatory encapsulation. Such information could support the comparative assessment of prosthetic materials, mesh architectures, and host tissue responses and may contribute to the development of materials designed to promote more favorable biological and mechanical integration [36]. However, the diagnostic, prognostic, and therapeutic relevance of these descriptors remains to be established. Before AFM-derived parameters could be considered for clinical application, they would require validation in larger prospective and preferably multicenter cohorts using standardized protocols for specimen collection, preparation, hydration, acquisition, and data processing. Reproducibility should be demonstrated across operators, instruments, and laboratories, and AFM-derived parameters should be correlated with quantitative histopathological findings and clinically relevant outcomes such as recurrence, chronic pain, infection, fistula formation, and mesh contraction. Only after such validation could clinically meaningful thresholds or prognostic associations be considered.

The principal contribution of this study is the demonstration that clinically well-integrated and pathological human mesh–tissue interfaces processed under identical experimental conditions show distinct histological patterns and differences in specimen-level AFM surface parameters. Pathological specimens exhibited reduced collagen continuity, greater fibro-inflammatory heterogeneity, higher surface-amplitude and extreme-height parameters, altered surface-height distributions, and less clearly defined directional organization. This exploratory framework may support future investigations linking tissue architecture and nanoscale surface organization with specific clinical outcomes, including fistula formation, recurrence, chronic pain, infection, and mesh contraction.

## 5. Study Limitations

The study included only seven independent biological specimens per group. The 64 AFM scans analyzed per specimen represented technical subsampling and did not increase the number of biological replicates; therefore, the complete numerical separation between groups requires validation in larger cohorts.

The study groups were not formally matched for implantation duration or mesh material, and the pathological group included both fistulizing foreign-body granuloma and hernia recurrence, precluding subgroup analysis. These factors may have contributed to the observed differences.

Histology was qualitative and did not include formal scoring, quantitative collagen analysis, or immunohistochemical characterization. Consequently, direct quantitative correlations between the severity of histological alterations and individual AFM-derived parameters could not be established. Incomplete information on mesh composition and architecture also prevented material-specific comparisons. Future prospective studies should incorporate standardized histological scoring and targeted immunohistochemical characterization, including CD68/CD163, α-SMA, CD3/CD20, and TGF-β, to enable quantitative histology–AFM correlations and better define the cellular and profibrotic mechanisms underlying the observed morphological patterns.

AFM measurements may have been influenced by RNAlater^®^ transport, partial hydration, progressive moisture loss during air-mode imaging, contact-mode tip–sample interaction, local tissue deformation, and filtering procedures. Although these conditions were standardized across both groups, they may affect the absolute values of the measured topographical parameters.

Accordingly, the findings should be regarded as exploratory comparative observations requiring confirmation in larger, standardized studies.

## 6. Conclusions

Pathological mesh–tissue interfaces exhibited more pronounced fibro-inflammatory remodeling, reduced collagen continuity, and greater surface heterogeneity than well-integrated specimens. At the specimen level, the pathological group showed higher Sa, Sq, Sku, Sp, Sv, and Sz values and lower Ssk values.

Histology and AFM provided complementary morphological information at different spatial scales. These findings support the use of AFM as an exploratory tool for characterizing mesh incorporation, although larger studies are needed before AFM-derived parameters can be considered diagnostically or prognostically relevant, or their relevance to biomaterial development can be established.

## Figures and Tables

**Table 1:** Patient-level clinical and surgical characteristics of the analyzed mesh–tissue specimens.

Specimen	Age (Years)	BMI (kg/m^2^)	Mesh Material	Implantation Duration (Months)	Indication for Reoperation/Mesh Removal	Infection/Contamination
P1	56	22	PP	7	Hernia recurrence	No
P2	45	21	Unknown	8	Foreign-body granuloma with fistula formation	No; cultures sterile
P3	42	19	PET	6	Foreign-body granuloma with fistula formation	No; cultures sterile
P4	65	23	PP	7	Foreign-body granuloma with fistula formation	No; cultures sterile
P5	50	22	PP	6	Foreign-body granuloma with fistula formation	No; cultures sterile
P6	63	21	Unknown	9	Hernia recurrence	No
P7	58	20	Unknown	7	Foreign-body granuloma with fistula formation	No; cultures sterile
R1	57	21	PP	14	Hepatic laceration	No
R2	48	20	PP	36	Gastric cancer	No
R3	59	21	Unknown	16	Sigmoid colon cancer	No
R4	65	22	PET	48	Uterine fibroid	No
R5	53	20	Unknown	13	Splenic laceration	No
R6	66	21	Unknown	48	Uterine fibroid	No
R7	45	19	Unknown	20	Perforated peptic ulcer	No

Note: P1–P7, pathological explants; R1–R7, well-integrated reference specimens. All meshes were implanted in the retromuscular plane and fixed with sutures. “Unknown” indicates that the mesh material could not be reliably retrieved from the available medical records. Abbreviations: BMI, body mass index; PP, polypropylene; PET, polyester.

**Table 2:** Specimen-level comparison of AFM-derived areal surface parameters between well-integrated reference and pathological mesh–tissue interfaces.

Parameter	Well-Integrated Reference Group, Median [IQR]	Pathological Explant Group, Median [IQR]	U; Exact *p*-Value
Sa, nm	6.655 [6.305–6.742]	13.060 [12.920–13.145]	0; 0.000583
Sq, nm	8.387 [8.270–8.763]	27.930 [27.515–28.200]	0; 0.000583
Ssk	0.4063 [0.1898–0.5118]	−0.2027 [−0.2159–−0.1973]	0; 0.000583
Sku	4.038 [3.097–4.192]	31.820 [31.435–32.070]	0; 0.000583
Sp, nm	44.20 [43.97–44.78]	304.10 [290.45–305.80]	0; 0.000583
Sv, nm	22.90 [22.71–23.12]	285.30 [284.70–286.15]	0; 0.000583
Sz, nm	67.13 [66.85–67.90]	590.30 [580.60–591.00]	0; 0.000583

Values are presented as median [interquartile range]; *n* = 7 independent biological specimens per group. Each specimen-level value represents the mean of 64 AFM scans. Between-group comparisons were performed using the exact two-sided Mann–Whitney U test. The reported U statistic represents the smaller of the two group-specific U values.

## Data Availability

The data presented in this study are available on reasonable request from the corresponding authors. The data are not publicly available due to patient privacy and ethical restrictions.
